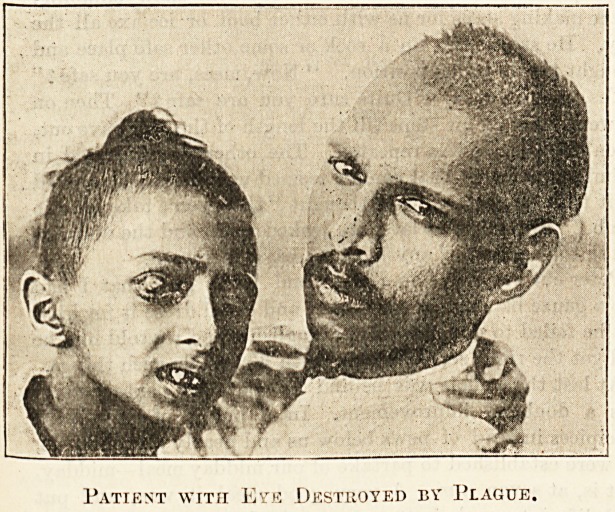# "The Hospital" Nursing Mirror

**Published:** 1899-08-12

**Authors:** 


"The Hospital" Nursing Mirror.

Being the Nursing Section of "The Hospital."

[Contributions for this Section of "The Hospital" should be addressed to the Editor, The Hospital, 28 & 29, Southampton Street, Strand, London, W.C., and should have the word "Nursing" plainly written in left-hand top corner of the envelope.]

### Notes on News from the Nursing World.

### ROYAL NATIONAL PENSION FUND.

The reception of the Pension Fund Nurses at Marlborough House has naturally brought the topic of provision for a nurse's age very much to the front, and many have asked how and in what manner the Pension Fund offers greater advantages than the many excellent insurance offices. The fact is that there are few offices that care to undertake annuity work because it is very troublesome, and therefore relatively expensive; that none of them will give bonuses on the earnings of their society or annuity policies, this advantage being reserved for life policy-holders; and that neither will any of them return the premiums in full if the holder of either life or annuity policy wishes to withdraw. As a rule the failure to pay a premium causes the policy to lapse. If any nurse want to know what course the National Pension Fund for Nurses pursues in such a case she should send for a prospectus to the Secretary of the Fund, at 28, Finsbury Pavement, E.C., and she will find, first, that all the work is annuity work, and that therefore annuity policy-holders reap to the full all advantages; secondly, that any profit accruing to the Fund through good management is divided periodically amongst the policy-holders in the shape of bonuses; and, thirdly, that a policy-holder can withdraw if she wishes, and receives not only her premiums back but interest on them as well.

### THE MEMORIAL TO HOSPITAL NURSES IN HONG KONG CATHEDRAL.

There has been placed in the north transept of St. John's Cathedral, Hong Kong, a stained-glass window in memory of the two nurses who died last year. Here is a description of the subject of the window. In the upper portion are two female figures, each of whom seems to combine the characteristics of devotion and work which were severally displayed by Mary and Martha of Bethany. One, on the right of the picture, is looking upward to the calm face of the Saviour, as though seeking the direction of His will: the other is in an attitude of service administering the cup of relief to a dying man. Over them, but unseen by them, on the left, is an Angel of Reward bearing for them the Martyr's palm. Above all is an emblematic crown. The whole picture very beautifully illustrates the words: "Inasmuch as ye did it unto one of these . . . ye did it unto Me." In the lower portion of the window are two angelic figures, one carrying a crown, and the other a "book," and bearing respectively on a scroll the words, "I will give thee a crown of life," and "I will give them an everlasting name." The names of the two nurses, with the dates of their deaths, are in the window. The work has been beautifully executed from a very artististic, appropriate, and effective design. Under the window there is a brass plate with the following inscription: "In loving memory of Elizabeth Frances Higgin and Emma Gertrude Ireland, who died in 1898 of plague contracted on duty. They were for nearly eight years members of the nursing staff of the Government Civil Hospital, Hong Kong. This window was placed in St. John's Cathedral by the community of Hong Kong."

### HOSPITALITY TO NURSES.

It was a kindly act on the part of Mr. and Mrs. Cosmo Bonsor to invite the staff of Guy's Hospital to Kingswood Warren. About two hundred nurses accepted the invitation, part of them going one day last week, and part the week before. Both days were exceptionally fine, and special trains conveyed the guests to and from London Bridge. On arriving at Kingswood Warren most of the party walked up from the station to the house, some availing themselves of the carriages waiting for them. Those who drove, together with Mrs. Bonsor and family, watched the pretty picture made by the others in their cool lilac prints, and snowy caps and aprons, wending their way up the hill to the terraces near the house. After light refreshments had been partaken of, the nurses wandered through the beautiful woods and gardens of the popular treasurer, many visited the farm, stables, &c., and some enjoyed tennis, croquet, and rounders, while others rested under the trees enjoying the music of the Blue Hungarian Band, and the lovely country air. During the afternoon tea was handed round in the garden, and in the evening the guests assembled on the lawn, where a cold collation was laid in a marquee on both occasions. Before the return journeys were made hearty votes of thanks were accorded to Mr. and Mrs. Bonsor for their unbounded hospitality.

### MATERNITY WORK AT GUY'S.

The midwifery training obtained at the Guy's Trained Nurses' Institution is now materially assisted by the nurses being enabled to do some work in the small maternity ward which forms part of the big gynæcological ward, "Queen Victoria." In this annexe of Queen's (as the big ward is tersely called) nurses who are being trained in midwifery in the district have six weeks' ward work as well. The rest of their time is spent in the district under the supervision of the district midwife, and, when necessary, of the obstetric physician from the hospital. The district worked from Guy's is a very large slice of the Borough, and Guy's nurses who go in for midwifery have every opportunity of receiving thorough training, especially now that ward work is a definitely included portion of it.

### THE BEAUTIFICATION OF BROMPTON JEWISH CEMETERY.

Nearly two years ago attention was first called in these pages to the deplorable condition of the disused Jewish burial ground at Brompton. We learn, with much satisfaction, that though expectations which were raised shortly afterwards were not fulfilled, the cemetery has now, by the munificence of a benevolent lady whose parents are buried there, been put into a thorough state of repair. Moreover, in order to complete the good work, a committee has been formed to renovate the tombstones, which, as we observed in October, 1897, are in a very dilapidated state, and to keep them in proper condition. The committee are appealing to those who have relatives lying in the cemetery to furnish the necessary funds, and we do not doubt that their appeal will be successful. To the patients and nurses at the Chelsea Hospital for Women, in whose interest we originally took up the matter, the renovation of the burial-ground is a most welcome change. The outlook has already ceased to be dismal, and when the entire improvement has been carried out the place, instead of having a depressing tendency, will form quite a pleasant oasis in the wilderness of bricks and mortar.

### UNTRAINED SOLDIERS AS NURSES.

Our attention has been called by an Indian correspondent to an important article in the *Civil and Military Gazette*, published at Lahore, on the subject of the nursing provided for sick soldiers in Indian military hospitals. Our contemporary shows that it compares very badly with the nursing in England, in Egypt, and in the Colonies, and proceeds: "The hospital orderlies are soldiers from the various corps in the garrison. They are more frequently sent for duty in hospital because they are not particularly wanted in barracks than for any special taste for nursing which they may have exhibited. With the exception of a brief garrison course on first-aid, they are totally untrained. It is to these men that severe cases of illness have to be entrusted. There are, of course, in some of the larger station hospitals two and, less frequently, three sisters of the Indian Army Nursing Service. These ladies do the most excellent work, and the only pity is that there are so few of them." The *Gazette* doubts, however, "whether in any case the nursing of soldiers in a military hospital is suitable work for a lady," and says that the nursing sisters are only useful, as now employed, for purposes of supervision; but it nevertheless very properly protests against "the system of entrusting the nursing of the terrible illness of the tropics to almost totally untrained soldiers." As a remedy it advocates the introduction of the Royal Army Medical Corps into Indian military hospitals, and as the article has attracted considerable attention, something in the shape of reform may be looked for. That it is urgently needed is only too obvious.

### THE NURSES AT CAMBERWELL INFIRMARY.

It is improbable that eight nurses would have sent in their resignations unless they had solid grounds for discontent. The vice-chairman of the Board of Guardians frankly says that in his opinion the discontent is due to the fact that the nurses have been reported for most frivolous matters, and he mentions as a case in point that one was reported "because a 'bus conductor raised his hat to her." During an animated discussion on the subject another member declared that "the girls' sweethearts were at the bottom of the trouble;" a third averred that "the nurses' quarters swarmed with rats, which so frightened them that they left;" while a fourth attributed the unsatisfactory position to the conduct of an official, whom he described as a "martinet." In any event, it has been wisely decided that every complaint brought against a nurse by this functionary is to be threshed out before the committee. But, meanwhile, what about the rats? It may not be easy to ascertain whether sweethearts are "at the bottom of the trouble," but the accuracy of the statement about the rats can surely be tested at once. There are things which nurses must put up with as best they can, but it is too much to expect them to live in quarters pervaded by rats.

### THE QUESTION OF HOLIDAYS.

A question of some importance was raised at a meeting of the directors of the Arbroath Infirmary the other day. The matron of the convalescent home had been offered for her holiday the month of October, the last week of December, and the first week of January. She cannot, it appears, have a holiday in summer because the convalescent home is full at that time of the year, whereas in the winter it is generally empty; and, of course, to this, we assume, she cheerfully submits. But she objects to the division of her holiday on the ground of the expense it entails. The objection was supported by Dr. Duke, who said, "It was unfortunate that a person with a small salary should have to take holidays at two different times in one year instead of getting them altogether;" and we entirely agree with him. Moreover, the matron complains that during the last two years the Weekly Committee have twice changed her holidays. They may have had good reasons for doing so, but they should be able to arrange to let her have her full holiday altogether, especially as, according to Dr. Duke, her place is taken in her absence by a very competent, experienced, and praiseworthy person.

### A CYCLE PARADE ON BEHALF OF A NURSING ASSOCIATION.

The cycle parade at Malvern, which last year was one of the events of August, will not be less popular this year, because the amount realised is to be given to the establishment of a trained nurse in the parishes of North Malvern and Cowleigh. Last year the committee were able to hand over £25 to the Rural Hospital and Provident Dispensary, and little doubt is entertained that the sum available for the Nursing Association will be larger. A local organ says: "Almost continuously throughout the year appeals are made to the congregations of our churches and chapels to assist in the furtherance of foreign missions, many of which are no more laudable than the local nursing associations. Large amounts are raised on these occasions, and yet sometimes we hear of local charities being inadequately supported." The committee of the cycle parade deserve credit for doing their best to remedy this state of things, and since the need of a nurse for North Malvern is freely admitted, her appearance is not likely to be much longer delayed.

### THE COMPENSATIONS OF DISTRICT NURSING.

The chairman of one of the great London hospitals said the other day that "tram conductors do not work so hard as the nurses of Queen Victoria's Jubilee Institute, who, moreover, are expected to accommodate themselves to any domestic arrangements made by the family of the patient." But there is another and a brighter side, and it is the testimony of many that no member of the nursing profession can find more compensations from her work than the district nurse. "Not long ago," writes a correspondent, "I was speaking to one of the Queen's nurses whose district lies in a remote country village. She had been a private nurse when she first embarked upon a career of her own, and now she said nothing would induce her to return to the work, though it is so much more highly paid. Most of her district visiting is done on her bicycle, and when her day's work is over she is her own mistress in the comfortable lodgings provided for her. The local committee are most kind, and relieve her of much worry, though they themselves, good souls! bother her not a little by the persistent way in which they 'look after' her. District work in a large town—which she had experienced before obtaining the rural appointment—she explained, was naturally much harder and more trying that her present occupation. Still, she repeated, 'Go back to private nursing? Never!'"

### OUTSIDE ACCOMMODATION FOR INFIRMARY NURSES.

The reply given by the St. Olave's Guardians to the Local Government Board respecting the provision for nurses is not altogether satisfactory. As the result of a visit paid by a lady inspector to Rotherhithe Infirmary, who reported that the sleeping accommodation for nurses was insufficient—eight nurses occupying one dormitory, and not even separated by curtains—the Local Government Board communicated with the Guardians. The Clerk has been instructed to inform the Board that "the Guardians had been so impressed with the want of proper accommodation for nurses that they have allowed several to live outside, paying them a weekly allowance in lieu of lodging." But outside accommodation is only a temporary and indifferent solution of the difficulty. It does not seem to be the intention of the St. Olave's Guardians to deal with the question in the proper manner, namely, by the provision in the Rotherhithe Infirmary of the accommodation required. For example, in her report, the inspector of the Local Government Board stated that on the occasion of her visit some of the nurses were trying to rest during the dinner hour, but "there was neither quiet nor privacy." Living outside does not meet this difficulty, and it creates others.

### THE IDENTITY OF "LADY GRISELDA."

In reference to the note in last week's "Nursing Mirror" headed "Aristocratic Hospital Nurses," a sister informs us that "she worked in the same hospital with the Lady Griselda, who is the daughter of the Dowager Countess of Airlie, not Aislie, and sister of the present peer." This settles the question of identity about which our contemporary was somewhat vague.

### THE NURSES' HOME AT THE LEOMINSTER COTTAGE HOSPITAL.

The handsome new cottage hospital which was recently opened at the little town of Leominster, in Herefordshire, by Lady Rankin, includes an excellent nurses' home, containing admirable provision for the inmates. A corridor at the rear forms the connecting link between the home and the main building. Care has been taken not only to make the interior useful, but the exterior attractive. Thus, the elevation to the street is effective in execution, being built of red brick, terra-cotta panels, and moulded courses throughout, with the large bay windows of the sitting-room carried up and roofed over with overhanging half-timbered ornamental oak gables, while the timbers are left in their natural colour, harmonising with the general elevation and the style of the design. All the roofs are covered with red Bromley tiles, and the home is in every respect worthy of the hospital.

### THE MATRON OF LUTON BUTE HOSPITAL.

The Committee of Management have signified the deep regret with which they received the announcement of the resignation of Miss Babcock, who has been matron of the Luton Bute Hospital for eight years. Several members declared that during the whole of the time Miss Babcock has discharged her duties in the most faithful and successful manner, and the Mayor gave practical effect to their feelings by proposing that a subscription list should be opened with a view to presenting her with a testimonial. A number of subscriptions were at once announced. Miss Babcock relinquishes her post on October 10th, and the question of the appointment of her successor has been remitted to a special committee of the hospital, consisting of two members of the House Committee, two of the General Management Committee, and two members to be nominated by the medical staff.

### BICYCLING FOR PRIVATE NURSES.

The private nurse often bemoans her fate that, as a rule, she cannot have many bicycle rides. There is, however, another side to the question; also an entirely different view, as seen by the patient. "I have just heard," writes a correspondent, "of a nurse in charge of a serious private case, who by gratifying her desire to spend her off duty time on a bicycle met with disastrous results. She was not an expert bicyclist, and, against the wish of everyone else, betook herself off alone for a ride in the country. When she had gone some little distance, upon turning a bad corner, she came into collision with a hay cart. With much force she was thrown to the ground. The wheels of the cart passed over her, causing a compound fracture of one leg, and a dislocated ankle. The bicycle was smashed to atoms. Luckily, a doctor happened to be at hand just after the accident. Subsequently, he went with the victim to a London hospital, a distance of 23 miles, and all the time the poor patient was minus a nurse."

### SHORT ITEMS.

Subject to the sanction of the Local Government Board, the Brighton Board of Guardians have decided to subscribe 10 guineas a year to the Brighton branch of the Queen Victoria Jubilee Nursing Institute.—It is rumoured that half the sum of £7,500 required to liquidate the debt on the National Refuges for Homeless and Destitute Children has been subscribed as the result of an urgent appeal, called "Save Our Property Fund," by Mr. Henry G. Copeland, finance and deputation secretary. The remainder, it is hoped, will soon be forthcoming.—On Wednesday, Princess Henry of Battenberg opened the new block of the Royal Hospital for Consumption, at Ventnor.—There has been quite an epidemic of marriage among the nurses of Hongkong. During the last few months five or six of them have been married.

### Gynæcological Nursing.

By G. A. Hawkins-Ambler, F.R.C.S., Surgeon to the Samaritan Free Hospital for Women; Assistant Surgeon to the Stanley Hospital, Liverpool.

(*Continued from page* 246.)

### Pessaries.

Pessaries are used for the mechanical support of the internal organs, when, of course, they are introduced by the surgeon. But medicated pessaries are also frequently used, and they are of the same shape as the suppository but much larger. They have to be passed into the vagina and pushed well upwards and backwards with the finger. It is a good rule for the patient to wear a diaper afterwards, as some of these applications cause free discharge. It is occasionally necessary to remove a pessary inserted for mechanical support. In this case, after lubricating her finger, the nurse will pass it inside the vagina and hook it within the rim of a ring pessary, or in the lower bar of a Hodge, and withdraw it slowly and gently and in a backward direction, so that the posterior part of the vaginal wall be stretched, and there will not be painful pressure on the sensitive anterior portion.

### Personal Hygiene.

Before she can properly take care of her patient, the nurse must learn how to take care of herself. Apart from the fact that one's health is a very vital factor in the quality of the work one does, and that we cannot stand the strain of long hours and anxious watching unless we have robust health—which also goes to the making of an even temper—the nurse will be associated with women who are not only very susceptible to infection of all kinds, but who are themselves in a condition to infect not only the nurse but other patients with whom she is brought in contact. The nurse will, of course, know that she cannot long pursue a life of irregularity as regards eating, sleeping, and exercise without a serious effect on her health. In my opinion she requires eight hours in bed, with undisturbed rest, every day, and it is important that she should have an hour's outdoor exercise, which may be secured except under the rarest possible emergencies. It will make her come to her work fresh and more cheerful, and will be an agreeable change for herself and the patient. Leading, as she does, an indoor life, which is not always blessed with the most perfect hygienic surroundings, she is to be particular to avoid constipation. A warm bath should be taken at least once a week, and before every major operation; and a cold plunge bath or tepid sponge every morning will be found extremely beneficial. If she is out of condition it must be reported to the doctor in attendance. A nurse who is suffering from sore throat, chronic catarrh of the ears, nose, or eyes, or from any infectious or contagious disorder, is not fit to attend patients, to whom she may easily convey that or worse trouble. She should change her under linen as often as possible, and always before a major operation. It is a difficult matter to keep the skin of the hands in good condition, but it is not the less important to do this. Little cuts about the fingers, or sores beneath the finger nails, are openings for poisonous matters of all kinds, which she may obtain from patients and from soiled dressings. She will, therefore, take particular care of her hands. If they are chapped at all, an excellent application will be found in the ordinary lead ointment, diluted to half its strength with vaseline. A little glycerine rubbed into the hands at night will also keep the skin soft and pliable, and prevent the dryness and cracking, which is a source of much annoyance. She will thoroughly wash and disinfect her hands before and after dressing any patient, for she may convey infection to the patient, who may have, if not active centres of infection, wounds, raw surfaces, and discharges, that are excellent growing grounds for germs; which germs, if introduced by the nurse, may light up serious mischief; though it entails a little extra trouble, this constant washing of the hands is a vital necessity. A good nail brush should be kept handy. It should never be used very long, and if kept soaked in a solution of carbolic acid, 1 in 40, there will be less danger of irritating sores coming beneath the nails if the bristle happens to prick them. A brush, if well made, of strong fibre, and not too small, will also be less liable to scratch the hands.

The nurse will always remember the invisible enemy which lurks everywhere, and especially in the sickroom, is liable to be of a virulent and destructive nature. By preserving her own health, she will be more able to resist the chances of local poison and keep clear of contagion.

There are special precautions to be taken in some cases of unusual virulence. Patients suffering from specific disease are particularly dangerous. The nurse must on no account dress them or come into contact with any of their discharges if her hands are not perfectly sound, and she must be careful to use absorbent wool that can be immediately destroyed, or towels that can be disinfected and boiled, for such cases. If she were to rub her eyes, for example, with fingers not perfectly clean after dressing such cases, she would in all probability set up destructive inflammation or arouse constitutional disease. The smallest crack or sore must be protected and watched till healed. If the nurse has wounded herself with anything unclean she must wash the wound under a current of water, and either touch it with a stick of caustic or with a drop of nitric acid if the infection be considered serious, or wash thoroughly with carbolic lotion.

In the course of a little time all these things are done as a matter of routine, but until they have become reliable routine the nurse must exercise constant, though not fussy, care, and cultivate what has been well called "the antiseptic conscience."

### The Patient.

When you are placed in charge of a patient you must not concentrate your attention on the particular disease for which she is being treated to the exclusion of everything else. There are many points you can observe which will be of the highest value to the doctor, to whom the patient's general condition is as important as the local trouble, and even in apparently small matters they may have the greatest interest. In a grave operation most minute care is taken by all concerned, but in apparently trivial ones it is not uncommon for the general health to be considered with less gravity. You will sometimes find patients die after a minor operation, such as restoring the perineum or something of less severity, because the patient has had Bright's disease, which has not been recognised, or some serious constitutional disturbance. Being with the patient constantly you see things to which you may draw the doctor's attention, and will make a point of noting them. If she were suffering from cough or were expectorating you would observe the sputa. If the urine were at all different from the normal, either in quantity or in appearance, you would also notice it. The patient's temperature would be taken as a matter of routine, and you will sometimes be surprised to find it running fairly high, without any obvious reason, until a more detailed examination has been made. The woman may be actually suffering from phthisis or from some fever, unknown to any of her attendants. I have known an abdominal section performed on a woman who developed scarlet fever next day, and infected ten or twelve patients in the hospital within a few days. Observation of the temperature would put you on guard for any such condition, and your ordinary attentions to the patient would show you the condition of her skin, whether there were any rash or unusual heat, or dryness, or tendency to sweat; and though in a majority of cases you will find little to note, there will be occasions when your observations will be of infinite value.

### A Year's Plague Nursing in India.

By a Sister.

### THE ITALIAN CURATIVE SERUM.—A TRAGEDY AT THE MAHRATTA HOSPITAL.

It was at the Arthur Road Hospital that I saw the Italian curative serum (Lustig's) tried frequently on patients in the very early stages of plague. It was not considered to be of much use if the patient had been ill for more than two days. The serum was injected in fairly large quantities, and sometimes twice a day, or only once, according to the condition of the patient. This continued for several days; the amount used at one time was from 5 c.c. to 25 c.c. There was no ill effect from the serum, which was absorbed almost directly, and the puncture covered with collodion. In my own experience I have only known of two cases where abscesses formed after injection, and these were proved to be caused by the hypodermic needle not being perfectly clean. Now as regards the efficacy of the serum. There is every reason to believe it may do good in time. The effect on the temperature, pulse, and respiration is most marked, but I have seen many failures as well as some satisfactory results.

Patient with Eye Destroyed by Plague.

Here again we tried the treatment of hot air and vapour baths, which no doubt had the usual sedative result, and it was very comforting to find a restless, delirious patient soothed into a quiet sleep after his hot air bath, but I cannot say I saw any further good result than this. So many of our plague patients had also suffered severely from famine, so that the poor creatures had very little strength to battle against such an awful disease. Their wasted condition was too pitiable.

In mentioning the complications to be expected in plague I only quoted those most frequently seen, but there were many instances where total blindness was a marked and distressing feature for some time. I have seen instances of this and of total recovery of sight as the patient grew stronger and better. It is most common in children. There are also cases of total loss of sight of one eye, and frequently the attention to the eyes of our patients was one of our chief cares. Then, again, the halting, stammering speech so typical of plague is known to end in complete inability to articulate a word, speech only returning after complete recovery. Patients were often able to walk about and appeared almost well, yet found great difficulty in pronouncing their words and regaining their natural voice.

By this time most of the natives were quite reconciled to English ways and treatment, and it was really wonderful to observe how much our ward assistants had improved, and what very excellent nurses some of them became, especially the ward boys, whom it was a pleasure to observe making the beds of helpless patients, feeding them, &c., in quite a "trained" way. They were very patient, considering how long their hours on duty were and how trying their work. They certainly ran a very terrible risk in attending on the poor helpless people, and many of them contracted and died of the disease in the discharge of their duty.

There were several plague hospitals in Bombay, and I must refer to the sad tragedy enacted in the "Mahratta Hospital," which was open to a certain caste of natives, and which was most popular owing to the freedom they were allowed, and also to the choice of treatment, English or native, whichever they preferred. Everything had been going on happily for some months when the head native doctor, who was very much liked and was in charge of the hospital, contracted plague and died from it. Very soon another doctor got it, and he died; then again a third, and a fourth! Later on, two of the dispensers and two ward boys all fell victims to it and died! And so actually within three weeks the whole of the medical staff was swept entirely away, and there was not one single doctor or dispenser alive who had been working there so short a time previously. Naturally, the awful event cast a great gloom over the hospital. It was most distressing for the nurses to see all these men, their willing fellow workers, dying one after another, and it caused great alarm amongst the natives. Moreover, no new doctor cared at first to take up what appeared to be a most fatal appointment; however, they were soon procured, and I must here add, in admiration of the native staff, that not one of them deserted his post. The sanitary authorities made a careful investigation of the hospital, and improved the ventilation, and the cause of infection again appeared to be the finding of dead rats in the flooring of the office and dispensary where the poor victims so often sat.

The climate of Bombay all through the summer was most trying, but I think that most of the nurses kept fairly well and were happy.

We in Arthur Road Hospital enjoyed our work there, which was not at all too heavy, and later on towards November plague had decreased so considerably that I felt I could leave, after completing my year's work, with the happy feeling that things seemed so much better and I could well be spared. Accordingly, on November 25th I had my resignation sanctioned by Government, and my plague nursing was at an end. I was really sorry for many reasons to leave. I liked the work, though it was very sad and at times hard, and perhaps unsatisfactory, but still I liked it, and had it not been that my health was not very good and that I felt the effects of the long Indian summer and needed a change, I would gladly have stayed on for another year. Indeed, I hope that later on, should any more nurses be again required, I may be again able to go out. Judging from recent accounts this does not seem improbable, as the disease appears to be again increasing in Southern India. As it is, I am sure the natives have suffered dreadfully. Thousands have died, and unless things improve very much the population of India will soon have decreased to an alarming extent.

On November 26th, 1898, I sailed for England in the "Oceanic," and after a pleasant voyage was truly glad to find myself at home once more, none the worse for my year's plague nursing in India.

### Nurses Among the Alps.

### A HOLIDAY EXPERIENCE.

THAT we must do some really hard Alpine climbing has been our settled conviction from the time we first decided to come to Switzerland, but now we are here the question as to which peak is to be our first ascent has been a vexed one. It must be interesting, that is, it must afford us hard climbing and excitement—snow and ice and rock—but it must not be too difficult for a first ascent.

To-day we have heard a description of the Unter Gabelhorn which has made us exclaim, "That is the peak for us!" So this evening we interviewed our handsome muscular guide, Peter Anton Biener, who said, "Yes, it would be a good climb." Evidently though he did not want us to decide on the expedition unprepared for what was before us. We must know it was difficult, a long, tiring, steep snow climb, then rocks and precipices; he would not take two of us without a second guide, and if we did it we should have made the first ascent this year. Would we rather do the Ryffel Horn first? But nurses are not afraid of hard work or new experiences, and being fired with a great ambition we said, "The Unter Gabelhorn we will do," and Peter replied, "Very good." So it was fixed—to be called at half-past one to-morrow morning, to start at half-past two; then followed instructions as to apparel, food, &c., and we bade Peter good-night, leaving him to engage the second guide. The sunset was bathing the Matterhorn in a glorious pure golden light, which caught the other peaks as well, but L—— and I went to roost early.

Whatever is it in human nature which makes you wish to go and do a thing which you really don't want to do at all, a thing which frightens you so that you cannot sleep, and which there is no earthly reason you should do? The old man (is his name August?), who is well known in the world of Alpine climbers for his ability apparently to sleep all the winter and stop awake all the summer, called me before half-past one this morning. What a miserable time to turn out of bed, and how I envied the third member of our party, who had decided not to accompany us, and who was reposing comfortably in the second bed in my room! There was something very dreary about that early breakfast, with three candles to light the great salle à manger, and the little wizened August to dance attendance on us. In the adjoining pantry Peter and Joseph were packing our provisions into the "rück sacks." We had to wait in the hall until that was accomplished and August had procured us alpenstocks from the dark lower regions, and we thought it strange to be turning out at that time with two foreign men we knew so little about. At last all was ready, and the procession turned out solemnly into the dark night, while Cassiopeia shone brightly above us, and there was a dim grey-white shadow which, standing out against the deep dark blue, told us the Matterhorn was clear. It was difficult to see our path at first, and I tumbled straight into a stream. "Vet?" inquired Peter. "Not much," I replied, and found I was all right so long as I followed exactly the two darker shadows of my guide's muscular legs.

Very soon we began to be so glad we had come. There is, I think, nothing so impressive as nature before the dawn among the Alps, when the dark blue sky begins to brighten and glow in the east, and the great mountains take shape and substance against the blue, and the smaller stars fade, leaving only a few brilliant ones, which seem like heralds of the day, and over all there is a great and solemn hush as if mountains and snow and ice, heaven and earth alike, were waiting in silent worship for the coming of the sun. And as we got high enough to see the glorious Monte Rosa range it came, a golden light at first and then the rose, which caught the white snow and the few little soft clouds which hung about the mountains. It was too beautiful for words, and we could only gaze and adore. It is a tremendous privilege to see a sunrise on the Alps.

But even for that you must not halt for long. If you want to climb a mountain without discomfort walk as slowly as ever you like, but let that walk be steady and continuous. To be spasmodic is fatal. Don't talk more than is necessary. It is waste of breath. And so we plodded silently, steadily upwards through the pines out on to the bare hillsides, and the day came and the larks sang, and we with all nature were glad.

Two hours or more brought us to the snow on which we had to skirt a very steep hillside. Now I knew that in Norway such a place as that was what turned my head. A very steep, smooth snow incline and at the bottom I knew not what. Peter remarked, "I'll hold your hand," and did so, putting his arm behind him. And I internally said, "Take one step at a time and don't look down or around," and though I sympathised with the temptation of Lot's wife I did not fall into it or meet her fate.

Later on we were roped and had first a very nice stretch of snow walking to do; the snow was crisp and firm, and we got over it gaily. Then came the couloir. And this meant three-quarters of an hour or so of very stiff snow climbing, Peter making steps for us with either boot or ice-axe all the way. He stood firm on a rock or some other safe place and brought me up to his position. "Now, mees, are you safe?" "Yes, thank you." "Quite sure you are safe?" Then on he went, cutting his steps till the length of the rope gave out, when the process was repeated. The other two followed in a similar manner. That couloir seemed unending. We looked at it from the bottom and thought "that won't take long to climb." Ten minutes later we looked again and the distance to the top seemed, if anything, to have increased.

Peter cut his finger on a rock, and I was glad that I had put a gauze bandage in my pocket and could dress it for him, but he failed to understand our English when we told him he had got the right sort of people with him to do such things.

At last the couloir was behind us, but our next position was a doubtful improvement. In a pulpit of rock, with precipices instead of pews below us and nearly all round us, we were established to partake of our midday meal—midday, that is, at seven a.m. A very good meal it was, and put fresh life into us before we began the last part of our ascent—the part L—— dreaded the most—the rocks and precipices. And when we began it she exclaimed, "I shall have to give in; I can't do it." Now, I did not know that it relieved her mind to open it like that, and that really she had no intention of giving in, and I was dismayed. However, Joseph undertook her training, assured her she could and would and should and must get to the top, so on she came.

I like rock. I think my ape ancestors cannot be very remote, for, give me handhold and foothold, and I enjoy myself. Precipices are not exactly pleasant, but, as in the operating theatre, so long as you are busy and have not time to think of the butchering that is going on on the table, you are quite calm and comfortable, so when your mind is occupied with finding the best hand and footholds you have no time to think that below the rocks your feet are upon yawns—space. I really think it was rather bad, and we could not have believed that we ever could have been taken up such places and over and round such dangerous rocks. And then when we reached the summit we must get to the further side of the cairn, and that meant striding right round a rock just as far as my two legs could possibly stretch themselves, and behind us was—nothing.

Then Peter took off his hat and shouted and shook hands with us and drank our healths, and then pointed out the mountains to us—the Bernese Oberland in the distance, and nearer to us the Weisshorn, Matterhorn, Breithorn, Monte Rosa, Mischabel Hörner, &c., &c. Oh ! it was glorious, and the blue Zermatt valley lay below us looking as if we could leap into it from where we stood. We rested for some time on the summit. Peter and Joseph chatted to one another in German, and we two girls fell to moralising, and agreed that if we were as good Christians as we were Alpine climbers we should do very well.

Then began the descent. Now I was sent first, Joseph followed, then L——, while Peter brought up the rear. It was worse climbing down those rocks than up; if you went face outwards you could see what you did not want to, if you went face inwards it did not always answer. "Which way, Peter?" "Straight down, mees." But straight down is over the precipice ! But no—we find there is a way; one step, two steps, and a third will appear as it is needed. So we don't question Peter's word, but go and find we can do it. Somehow we scrambled down, not always in the best style, very seldom elegantly, now and again with the whole of our weights on the rope, but anyway and always landing safely on a lower ledge. More rest and food and drink on the pulpit, and then the snow. That was fun ! It was soft now, and Joseph went first at a good pace, making big foot-holds very far apart, into which we leaped and plunged, sometimes sinking up to our knees and having difficulty in hauling ourselves out, or having to wait till a guide came and pulled us. How we laughed and slid and tumbled and enjoyed ourselves, and were quite sorry when we reached rocks and earth and grass again, though it was certainly refreshing to lie down by a stream of water and drink and drink and drink again. I laid down on the very brink, so that I did not have to move except to put my head on a level with the water whenever I wanted another draught. The others drank out of one glass by turns, and the guides did not agree with me that fresh cold water was infinitely superior to cold tea or "vin," or a mixture of both.

And so we reached the lower alps, down which we ran and jumped, not feeling in the least tired, and we landed once more at the Monte Rosa Hotel just as the lunch bell was ringing. We shook hands with our kind guides and thanked them very truly, and would recommend Peter Anton Biener and his brother-in-law, Joseph Taugwalder, to any man or woman who wants a guide in Zermatt.

Morals: 1. Take your duty step by step, and never try to foresee or understand the whole. 2. Trust is doing what you are told and not being afraid of the consequences. 3. If you make even a single step of your own it may not lead to disaster, but it does mean waste of energy. 4. Difficulties and dangers ahead seem overwhelming, viewed at hand they are always possible, very often are not difficulties at all. 5. The knowledge that you have a good guide, who knows the way and is absolutely reliable, is sufficient to make you comfortable in every emergency. 6. Life is absolutely, infinitely worth living.

### Minor Appointments.

SPITTLESEA INFECTIOUS HOSPITAL, LUTON.—Miss Elizabeth Downing has been appointed Staff Nurse. She has held appointments at Huddersfield Infectious Hospital.

### Presentations.

THE BERRINGTON NURSING ASSOCIATION.—Mrs. Elizabeth J. Beswick has been presented with a silver tea service, a silver-mounted biscuit box, and a silver cruet, subscribed for by the women of Ashton, Moreton, Luston, and Bircher on the occasion of her resignation of the post of Queen's nurse after seven years' work in the Berrington district, Herefordshire.

### A Nurse's Experience among the Pilgrims from Mecca.

TOR is a small mud village on the Arabian coast of the Red Sea. During the winter months there is nothing particularly interesting about the place, but from May to August the village presents a much more animated appearance. In that time the people who have performed their pilgrimage to the shrine at Mecca return to their respective homes, and before they are allowed to land in Egypt they are examined at Tor, in order to prevent the spread of any infectious disease, such as plague, cholera, or small-pox, which they might have contracted during their wanderings.

As each boatload comes down the Red Sea the passengers are landed at Tor. In readiness for them is a medical staff of different nationalities, one English nurse, a hospital, stores, and camps. As the pilgrims land they are isolated in the large enclosures, and all arrangements are made for their three weeks' quarantine. During that period each pilgrim is examined three times—on arrival, ten days later, and on leaving.

The nurse takes charge of the women, and their examination is by no means an easy task. An endless fund of tact and good humour is required, as they object very strongly, and have innumerable ways of trying to evade the authorities. The head medical officer must sometimes be called in to assist, but as it is entirely against an Arabwoman's sense of propriety to let any strange man see her, this is only done as a last resource. Lists are strictly kept, and in the end the pilgrims find they must submit to the ordeal. If there should be any suspicious cases which might turn out to be infectious they are carefully watched, and the hospital is always ready for isolation.

The camps cover a large space of ground, so the nurse rides about from one to the other on the donkey which is provided. Her tents are a little apart from the other Europeans in the camp, and she has her own black manservant to do her cooking and generally wait on her. She should bring a good supply of tinned provisions with her, as the food from the stores is very costly, and on days when there is not much to do her servant takes the donkey and goes to the outlying villages to procure fresh fruit, vegetables, eggs, and chickens. There are no other European women in the camp, so the nurse needs plenty of books and work to while away the time. A boat only visits the camp once a fortnight with news from the outside world.

The work is for a time very interesting, but as the pilgrims arrive less frequently, and there is little to do, the time hangs more and more heavily, until at the end of three or four months the nurse is very glad to return to civilisation, though not at all sorry to have had the experience and change.

### Marriage of a Nurse.

LAST week Miss Mary Selina Addams Williams, who only recently left the County Hospital, York, where she worked for the last six years, and where, as sister of the accident ward, she was most highly regarded by all who were brought into contact with her, was married to the Rev. C. A. Cherrington, eldest son of the Rev. A. C. Cherrington, vicar of St. Margaret's, Ladywood, Birmingham.

The bride was given away by her father. The Misses Jessie and Marcia Addams Williams, sisters of the bride, were the bridesmaids, and the Rev. H. S. Ratcliffe, Haigh, Wigan, supported the bridegroom as best man. The church bell was rung, and the bells of St. Mary's Church, Monmouth, also pealed out merrily at intervals during the afternoon. As showing the great respect in which the bride's family is locally held, two floral arches had been erected *en route* to the church, one at the commencement of the Redbrook Road, near the railway bridge, and the other, a smaller one, on the Wyesham Road. Both looked very pretty, and bore suitable mottoes. A reception was held later at the bride's home, and during the afternoon the happy pair left for their honeymoon trip, which is being spent in North Wales. The wedding presents were numerous and choice.

### Echoes from the Outside World.

### AN OPEN LETTER TO A HOSPITAL NURSE.

It has long been customary to give the recently-confirmed some little gift to remind them in the future of the solemn duties they have taken upon themselves the day of their confirmation; but, generally speaking, these gifts are confined to a Bible, a Prayer-book, or a religious work. However, the list of presents which were bestowed upon the young Duke of Albany last week makes one afraid lest the Royal fashion should be followed by commoners, and we shall have to look upon the confirmation of our young relatives as an occasion which demands an outlay as serious as a twenty-first birthday or a matrimonial alliance. The Queen, it is true, gave a Coburg and Gotha hymn-book and Prayer-book in German, but then she also added a costly silver tea and coffee service, with a crown and V.R.I. on one side, and a crown and the young Duke's initials on the other. The Prince of Wales, who is both godfather and guardian, gave the Prince a massive embossed silver bowl; the Duke of Connaught four silver dishes; Princess Christian a silver hot-water jug; Princess Louise a silver inkstand; and the servants at Claremont Place a pair of silver candlesticks. The Duchess of Albany's offering strikes one by its very simplicity—a German Bible, inscribed with the German motto, "Treu und feste."

Poor old Pope! no wonder he looks upon the recent gift of a French manufacturer as somewhat of a white elephant. His age is so advanced and his life is so valuable that it is not surprising he hesitates to risk his bones in a motor car, even though it be of the most superior make. If he has read the papers of late he will probably have been struck by the number of accidents which occur either to the machines themselves or, worse still, to the occupants, so that the chances of his driving about the gardens in his automobile carriage are slight. At present it is looked upon simply as a curiosity, and will probably soon get out of order for want of using.

Regular visitors to the Academy must have grown accustomed to notice on the walls, year after year, the pictures by Mr. Sidney Cooper, R.A. There is invariably a polished, highly-finished look about them, which at once distinguishes them from the more modern Impressionist style, and the animals always look exceedingly well-groomed. But few of you know, perhaps, that the painter of these farmyard scenes has passed his ninety-sixth birthday, and still does all his work without glasses of any kind! He was asked to what causes he attributes his long life. His reply is worth recording. "To God's providence and mercy, for I consider myself," he said, "a monument of His mercy; also, to a certain extent, to my own industry, and to my system of always living temperately, and living *out of London*; and I have drunk neither tea nor coffee for over forty years." Mr. Cooper went on to state that he is very regular as to the hours of meals, also in taking daily exercise, and that he is a great advocate of porridge, which he not only finds very sustaining—an ordinary experience—but he likewise maintains that he finds it provocative of good appetite. Now, the only fault which I have to find with the homely Scotch dish is that it begins your breakfast—and finishes it, too! But evidently it has not the same effect upon everyone.

Are you going to the seaside? And, if so, are you contemplating bathing? Because, if so, you may like to know the very latest on the subject of bathing gowns. If you wish to be in the swim—no pun intended—you must abandon the idea that a sedate, dark blue serge trimmed with white will suffice; it may answer the purpose, but it will never look *chic*. To look *chic* you must have the material brightened by something striking—black trimmed with orange, white with pale blue, dark blue with cerise, and, above all, the costume must be cut princess shape. This will necessitate the fastening coming down the back, and the consequent lady's maid to help you in and out of your bathing attire; but that is a mere detail. Then, shoes and stockings should be worn; they may be dark or bright in colour, as you prefer, but if you wish to be French your hose should be of black silk and your shoes should be white. Your cap must make a mark—bright green or turquoise blue, geranium-red or vivid orange, with perhaps a few lines of something paler to prevent the strong tints being too aggressive, and a bow of ribbon of the same colour tied round your neck, unless your neck be very plump, when you may dispense with this fashionable adjunct. If you think very much of your personal appearance a bathing fringe tacked firmly—very firmly, mind—inside your bathing cap will distinctly improve your beauty. Just one more reminder. If you wish your dress to look really nice be sure you have it rinsed in fresh water every time after using, and have it ironed carefully so that no obnoxious creases may remain. This, of course, can be done again by the lady's maid, the same individual who fastens up your princess bathing dress. If by chance you have to go to the seaside without a maid, why I advise you to wear a gown of a less ambitious character.

A very few years since nothing was considered too low for workhouse inmates. It was considered good policy to let them feel their degradation in every way; they were never allowed to forget that they had committed the unpardonable sin of being poor, and as long as they were given enough food to keep body and soul together the guardians felt that they had done the whole of their duty. Now, I rejoice to say, things are very different. Those who are deserving, even though they have the misfortune to be poor, are no longer obliged to wear a workhouse garb when they take their walks abroad; when ill they are no longer left to be nursed solely by incapable paupers who are too feeble to do more arduous work; and they are even permitted to go for a pleasure expedition if some kind soul will "pay the piper." Furthermore, quite lately the Poplar Board of Guardians have purchased two gramaphones, together with a selection of music to be played by them, for the amusement of the inmates of their workhouse. At Scarborough a lady and her pupils offered to present a piano for the entertainments to be given at the workhouse, and the offer, instead of being curtly refused, as it would have been some little time back, was cordially acknowledged by the guardians. Assuredly, we have advanced in some things.

Even the masculine mind has been struck by the fact that women's faces are no longer covered by veils. A man remarked to me the other day, "I am so glad to see that ladies are giving up veils. At last we shall be able to find out whether a complexion is real or not." I told him as nicely as I could that his politeness was as much at fault as his powers of perception. There is a decided revolt against veils at present because the weather is so hot that even the addition of a piece of tulle stretched across the face is to be avoided as causing a slight access of heat. But let Boreas blow in real earnest, and the lover of tidiness will be bound to put on a veil unless she wishes her locks to stray about in that fashion which is so artistic and charming in books and so hideous and unbecoming in real life. Then, too, the soft summer breeze fanning the face produces nothing but a sensation of pleasure. But when the biting frost is on the ground and the driving snow is in the air, those who object to rough cheeks and cracked lips will once more fly for succour to the friendly veil. I do not believe that either the manufacturers of "falls," as they are often called, or the vendors of them, need fear that they will not be in request later on in the season.

### A Book and its Story.

### THE COURT LADIES OF PAST GENERATIONS.

Mrs. Richardson, in the lives of her famous Court ladies,* has given to the public a very interesting series of historical character sketches of women not unknown to fame, and, by the courtesy of their noble descendants, has had access to private manuscripts and permission for reproduction of the valuable portraits and paintings by which the work is enriched. Over seventy engravings of leading men and women of their day illustrate and add to the value and interest of her record. She has performed her task in a singularly fair and impartial spirit, her desire being "to see through rancours and jealousies of memoir-writers and diary-keepers, and to make due allowance for the jocularities of letters intended for the eyes of those alone to whom they were addressed." By careful research into the circumstances and personal environment of her heroines, she has in many instances been able to give a more generous reading of a character than that generally received.

"The judgments which the world passes on women, and which historians, male and female, are so prone to echo, seem to me to err ever on the side of praising them for one great virtue and decrying them for one great vice. If, in characterising some of the prominent ladies of the Court of England, my descriptions go to prove that in times past there existed women, as many still exist and more will, who were other than what men directly made them, they will have helped to establish the truth that female character as well as male may be individual and distinct."

Certainly the perusal of Mrs. Richardson's characters leaves the reader in no doubt as to their strong and distinct individuality. With all their failings, the one of lack of directness of aim cannot be laid to their charge, whether in political or personal affairs, and if those very charms inseparable from beauty and high birth were not always used to highest ends, still they were after all very women, and in most instances of high principle as well as of strong character.

Their record begins with the redoubtable Elizabeth Countess of Shrewsbury ("Bess of Hardwick"), whose mania for raising magnificent buildings and ostentatious love of display is so well known. Hardwick Hall, that splendid pile of masonry familiar to all visitors to the peak district of Derbyshire, survives with Chatsworth as a memorial of her. The present palatial building has long outgrown the original and less pretentious edifice. Sheffield, Worksop, Oldcoates, and Bolsover were also finished in her lifetime. For sixteen years the earl and countess, whose domestic life was none of the smoothest, had as their captive guest the lovely Scottish Queen Mary. She was in their charge alternately at the castles of Tutbury, Wingfield, Sheffield, and Chatsworth. It is pretty clear that Queen Elizabeth had chosen the Shrewsburys as her custodians, having an intimate acquaintance with the character of "Bess," and her peculiar fitness, from her point of view, for the post of guardian. To do the countess justice, in spite of much intriguing to further the claims of her grandchild, Arabella Stuart, should occasion arise, to the English throne, she behaved well, with a certain measure of kindness and display of devotion to her guest. But the coming of Mary of Scotland into the Shrewsburys' household seems to have brought, as it ever did, to those who served her, shadow and unrest. Certainly, the harmony of the household was by no means enhanced by her presence. Later the Earl complains of want of "the appreciation of his sovereign for the faithful discharge of the onerous task of guarding Mary Queen of Scots."

* "Famous Ladies of the English Court." By Mrs. Aubrey Richardson. (Published by Hutchinson and Co., London.)

"When the tragic destiny of Mary Stuart was speeding to its consummation, an illness saved Shrewsbury from the horrible task of signing her death-warrant. But he was present at her execution. To this fact he points in the epitaph on his tomb in Sheffield Parish Church, as a conclusive proof that the scandalous accusations brought against him were never really credited."

"Before his death the remaining years of the poor earl's life were embittered by countless lawsuits brought by the countess, from whom he had separated, to dispossess him, during his lifetime, of the greater part of his property." . . . "All the trump cards of life had fallen from the great earl's hands. But the countess held hers to the end." The following paragraph sums up graphically her leading traits: "The tongue of persuasion, the actress-soul which gave her ever power to play a leading part, the astute mind, the unsensitive nerve, and above all, the unassailable belief that what Bess wanted and what Bess willed were the supreme needs and laws of existence, made her self-content invulnerable." She was four times married in spite of her masterful character. A prophecy ran that "So long as she kept building she would survive, and during a winter of hard frost, when her men were unable to work, she died."

In the mother of that accomplished statesman and poet, Philip Sidney, a very different personality stands before us. "The pretty, gentle creature, in rich Court costume, who, from her portrait on the walls at Penshurst, has looked down pensively on many later generations of Dudleys and Sidneys, and looks down still, is the Lady Mary Sidney." "A woman nobly born and courtly bred, she yet held it her dearer pride to be a faithful wife to her good lord." In the spring of 1578, Philip, writing to his father, Sir Henry Sidney, concerning the friends at court who were trying to make the difficulties of his post of Lord-Deputy to Ireland easier to him, thus speaks of his mother: "Amongst which friends, before God there is none proceeds either so thoroughly or so wisely as my lady, my mother." And then he paid her that tribute than which, perhaps, there is none more touching, as from a son to a mother, in all our English annals: "For my own part I have had only light from her." That is the Lady Mary's character, a nature which threw light; an illuminating presence. Only sweet reasonableness and dear unselfishness can clothe a woman in such rare effulgence. And hers was a nature in which strength was combined with sweetness and saving common-sense, which made her invaluable to her noble husband as a helpmeet in his public life.

No collection of notable Court ladies would be complete without the names of Sarah Duchess of Marlborough, of Lady Anne Clifford, the beautiful, eclectic Lucy Countess of Carlisle, and many others, all of whom receive impartial and adequate delineation from Mrs. Aubrey Richardson. Her book is most beautifully got up and leaves nothing to be desired on this point. From a literary point it fulfils thoroughly what it professes to do, and we wish all our readers the same pleasure in perusal which we ourselves have received.

### To Nurses.

In order to increase and vary the interest in the *Mirror*, we invite contributions from any of our readers in the form of either an article, a paragraph, or information, and will pay a minimum of 5s. for each contribution. All rejected manuscripts are returned in due course, and all payments for manuscripts used are made at the beginning of each quarter, *i.e.*, January 1st, April 1st, July 1st, and October 1st.

### Everybody's Opinion.

[Correspondence on all subjects is invited, but we cannot in any way be responsible for the opinions expressed by our correspondents. No communication can be entertained if the name and address of the correspondent is not given, as a guarantee of good faith but not necessarily for publication, or unless one side of the paper only is written on.]

### CASES GIVEN AWAY GRATIS.

"GIVE AND TAKE" writes: Frequently, when I have more cases than my own staff can undertake, I give cases to nurses working on their own account. It seems only fair that I should receive a percentage of the fees taken in such cases. I should be glad to hear from other superintendents what system they adopt in similar circumstances.

### THE NELSON HOME, MANCHESTER.

"R. N. P. F." writes: During last winter I had an opportunity of learning something of Miss Stewart and her home, for I was nursing a patient whose daughter-in-law was at that time undergoing an operation there. I remember how everyone spoke well of the institution, and it pained me not a little when I read the account in THE HOSPITAL of Miss Stewart's recent unpleasant experience. However, I congratulate her on her victory.

### PRIVATE NURSING INSTITUTES.

NURSE FLORENCE DORÉ writes to complain of the manner in which she was treated at one of these institutes. She says: I entered as staff-nurse at the rate of £35 a year, with laundress. On the day I started there I had the offer of four good posts, and because I would not break faith with the superintendent of the institute I let them slide and went to her. I was in the house a whole month without a case coming in, and I then gave a month's notice from June 30th. I afterwards had two mental patients—one for a single night and the other for two. The second patient declined to stay in the institute, and went to the house of a friend of mine in the same square. I had nothing whatever to do with her going, but on the same day (July 15th) the superintendent said to me, "I do not require your services after to-day." Nurse Doré proceeds to describe the accommodation as most inferior, to state that she was frequently left in the house alone in the evening, that male massage patients were received, and that she had to take legal proceedings to recover the amount she considered due to her. She claimed £2 and expenses, and received £1 14s. 6d.

### BREAKFAST IN THE BEDROOM.

"ONE WHO LIVES TO LEARN" writes: I should be glad to know if it is at all frequent in private houses to expect nurses to have their breakfast in the bedroom—either their own or the patient's, or, where they share a room, the common one. In one house where I nursed mine was brought to me in the dressing room, where I had slept and made my toilet, and where the window, therefore, had to be open. This was in winter. As it happened, my patient's room was very large, and having a fire, and the window having been open all night by her own wish, I could have it comfortably there, and did so, as she had no objection. The principle, however, is the same. At another house, where I slept in a small room with a full-grown girl, and the room was not allowed to be aired, my breakfast was on the first morning brought up with hers, on a separate tray. I carried my tray down bodily, telling the lady of the house that I did not mind where I had it so long as it was not in the room where we had both been all night, or something of that kind; but it needed a considerable effort to do this. I am curious to know if many nurses would have put up with the intended arrangement, and whether, indeed, it is looked upon as all in the day's work that one's ordinary instincts should be ignored in matters of the sort. I am not speaking of cases of infectious, or of very severe illness; and, of course, I know that "meals in a separate room" are nominally stipulated for by institutions for their nurses. But in actual practice, and where the patient is laid up and needs a nurse without being medically ill, what is "the sense of the House" on the question?

### NURSES AND BICYCLES.

THE MATRON OF THE MANCHESTER ROYAL EYE HOSPITAL writes: *Apropos* of the paragraph *re* a nurse's bicycle in your last week's issue, I should be glad if you would make known through your columns that Miss Gaskell, the vice-president of our ladies committee, recently presented the nurses of this institution with a bicycle, which is greatly appreciated. The fact of this being known may induce others to follow her example at other hospitals.

### LECTURES UPON PRIVATE NURSING.

"VERA" writes: I wonder whether it has ever struck others, as it has struck me, how very helpful it would be for nurses if during their time of training they received lectures upon private nursing. It is so frequently the case that a nurse is sent straight from ward work into a private house to nurse a case, and the change is in every way so great a one that the nurse is sometimes quite puzzled and at a loss. It is such totally different sort of work, and the surroundings are so unlike what for three years at least she has been accustomed to. Nowadays we hear complaints against nurses, and some people are extremely averse to having a trained nurse at their house at all. Surely this feeling might be modified if matrons could send their nurses out better prepared for what they may have to face in private houses. Private nursing is, as a rule, neither easy nor pleasant work, and nurses very often make their own lives less happy than they might be because they do not learn to adapt themselves to whatever household they may find themselves in. I think that if every hospital could arrange for a course of lectures on private nursing it would be an immense gain. Supposing that the matron herself is too busy a person to give such lectures, why should not a competent person, accustomed to private nursing, be asked to give courses of lectures on the subject? I believe that the profession generally, and also the public, would be greatly the gainers.

### Appointments.

STEWART INSTITUTION, PALMERSTON, CHAPELIZOD, COUNTY DUBLIN.—On July 20th Miss Eleanor J. Law was appointed Matron. She was trained at the London Hospital and subsequently held a staff appointment there. She has since been sister in charge and matron for three years at St. John's Hospital, London; matron at Mercer's Hospital, Dublin; and superintendent and matron at the Royal Berks Hospital, Reading.

BRITISH HOSPITAL FOR SAILORS, ALGIERS.—Miss Gertrude Ward has been appointed Matron. She was trained at St. Thomas's Hospital, and was afterwards a Queen's nurse in London. Subsequently she joined the staff of the Universities Mission to Central Africa, which she was obliged to resign this year on account of the climate.

CHILDREN'S BRANCH, METROPOLITAN CONVALESCENT INSTITUTION.—On August 8th Miss L. Blanch Pepper was appointed Lady Superintendent. She was trained at the Children's Hospital, Great Ormond Street, and has since held the posts of sister at Victoria Hospital, Chelsea, and assistant matron, Sheffield Royal Infirmary.

FOUNTAIN HOSPITAL, TOOTING.—Miss A. Thomas has been appointed Assistant Matron. She was trained at the General Hospital, Birmingham, and has since been charge nurse and also night superintendent at the Brook Hospital, Shooter's Hill.

BRIDGWATER INFIRMARY —On August 4th Miss Ellen Burr Rackham was appointed Sister. She was trained at the County Hospital, Lincoln, and has since held appointments as staff nurse and head nurse in the same institution. She has also been for a year district nurse at Barton-on-Humber.

KETTERING AND DISTRICT GENERAL HOSPITAL.—On August 3rd Miss Kate Steen was appointed Matron. She was trained at the London Hospital, and has since been sister of the female surgical ward at the Taunton and Somerset Hospital, Taunton, &c.

BURNLEY DISTRICT SANATORIUM.—On August 7th Miss Amy P. Girling was appointed Matron. She was trained at Bolton Infirmary, and has subsequently been night superintendent at the Victoria Hospital for Burnley and District and sister-in-charge of the male medical and surgical ward.

### For Reading to the Sick.

"I RECKON that the sufferings of this present time are not worthy to be compared with the glory which shall be revealed in us."—1. *Rom.* viii. 18.

Thou hast done well to kneel and say,
"Since He who gave can take away,
And bid me suffer, I obey!"
And also well to tell thy heart
That good lies in the bitterest part,
And thou wilt profit by her smart . . .
Nor with thy share of work be vexed;
Though incomplete and e'en perplext,
It fits exactly to the next.
What seems so dark to thy dim sight
May be a shadow, seen aright,
Making some brightness doubly bright.
The flash that struck thy tree—no more
To shelter thee—lets Heaven's blue floor
Shine where it never shone before;

—*A. Procter.*

Show me the path! I had forgotten Thee
When I was happy and free,
Walking down here in the gladsome light of the sun;
But now I come and mourn; oh set my feet
In the road to Thy blest seat!
And for the rest, O God, Thy Will be done!

—*J. Ingelow.*

**Reading.**

The sympathy of Jesus in suffering! Suffering is a deep mystery, and always will be, and yet we know this about it, we know that it is the school in which God often teaches the deepest and the most lasting lessons. Suffering is a sacred retreat, in which God would draw souls nearer to Himself. Suffering is the school in which the soul is often taught lessons, which it would never learn in any other way. It is something, surely, to face, just for one moment, the meaning of pain by the cross. People sometimes ask how, in view of all the pain and suffering which He permits, we can think of God as a God of love? If God be love, why does He permit so much pain, so much suffering, in the world? Well, whatever else may be said about it, this is certainly true, that the one end of all physical suffering is to draw the soul nearer to God. We do a direct wrong to God's love if we regard suffering as if it were sent by an angry God. It is something, as we try to recall the cross, to know that "though the Lord cause grief, yet will He have compassion according to the multitude of His mercies. For He doth not afflict willingly, nor grieve the children of men." God, ever remember, is a God of love, who would draw His children, by all means, nearer to Himself. Let us never therefore think of pain as if it were ruthlessly sent by some hard, unloving Being, who likes to see His children suffer.—*F. W. Isaacs.*

Teach me to live! 'Tis easier far to die—
Gently and silently to pass away—
On earth's long Night to close the heavy eye,
And waken in the glorious realms of Day!
Teach me that harder lesson—how to live,
To serve Thee in the darkest paths of life;
Arm me for conflict now, fresh vigour give,
And make me more than conqueror in the strife!
Teach me to live Thy purpose to fulfil!
Bright for Thy glory let my taper shine!
Each day renew, remould this stubborn will!
Closer round Thee my heart's affections twine!
Teach me to live, and find my life in Thee,
Looking from earth and earthly things away;
Let me not falter, but untiringly
Press on, and gain new strength and power each day.
Teach me to live! with kindly words for all,
Wearing no cold, repulsive brow of gloom,
Waiting with cheerful patience till Thy call
Summons my spirit to her heavenly home!

### Notes and Queries.

**The contents of the Editor's Letter-box have now reached such unwieldy proportions that it has become necessary to establish a hard and fast rule regarding Answers to Correspondents. In future, all questions requiring replies will continue to be answered in this column without any fee. If an answer is required by letter, a fee of half-a-crown must be enclosed with the note containing the enquiry. We are always pleased to help our numerous correspondents to the fullest extent, and we can trust them to sympathise in the overwhelming amount of writing which makes the new rules a necessity.**

**Every communication must be accompanied by the writer's name and address, otherwise it will receive no attention.**

*Medical Nursing Lectures.*

(164) Could you kindly tell me whether there is a book published entirely on medical diseases, where it can be obtained, and the price? I want something after the style of "McAdam Eccles' Elementary Surgery for Nurses," only medical lectures instead of surgical. I have not seen one advertised at any time, and the booksellers I have been to do not seem to know if there is such a thing. Laurence Humphrey I already possess. I should be greatly obliged to you if you could help me in this as you have done in many things in the past.—*B. K. T.*

Possibly the reply to "New Reader," under the heading "Knowledge" in last week's "Mirror," would meet your want.

*The British Soldier.*

(165) What measures ought a trained nurse to take who wants to nurse the English soldiers if there is war in the Transvaal?—*E. G.*

The British soldier is nursed in war by the Army Staff Corps, and by the sisters of the Army Nursing Service. Information as to how to enter the latter service may be obtained from the Adjutant-General of the Forces, War Office, Pall Mall, S.W.

*Poison.*

(166) Should I be in any danger of breaking our law if I were to sell one ounce of (Fowler's Solution) arsenecalis? I know it is poison, but it is for a certain disease, and is only taken in drops. My uncle sold it for over 13 years, and he is now dead. Some one has told me that if I were to sell it I should be liable to a fine for breaking the law.—*Sarah A. C.*

You would certainly break the law, as poisons are only permitted to be sold by qualified dispensers, and then under stringent regulations.

*Lodge Keeper.*

(167) I am anxious for my parents to obtain an appointment as lodge keepers at a hospital or home. Could you advise me as to best medium through which to hear of the same. My father is well educated, he understands gardening and carpentry, and seems well fitted for such a post. He is very handy and willing, healthy, is middle-aged, and has excellent references.—*Nurse W.*

Advertisements for persons suitable to such appointments are not infrequent in the "Mirror." An advertisement would probably bring more than one reply.

*Young Probationers.*

(168) Could you kindly tell me whether in any of the leading hospitals, either of England or Scotland, they receive probationers at the age of 20, and if so what premium is asked?—*Twenty.*

Only some children's or special hospitals receive probationers so young. We hear that the matron of the Flemming Memorial Hospital, Newcastle-on-Tyne, has a few vacancies.

*The Nauheim Treatment.*

(169) Will you kindly inform me in what places in Scotland the Nauheim baths (for the treatment of heart disease) are given?—*Alison.*

The so-called Nauheim treatment is being so rapidly introduced at many watering places that it would be best to inquire at any spa which it is proposed to visit whether it is in use there. At none of the spas of Scotland do the waters appear to be so like those at Nauheim as to render them specially available for the treatment, but the same is the case at many places where it is very efficiently carried out.

*County Medical Officers.*

(170) Miss C. C. is anxious to find out the names of all the county medical officers in England and of the towns where they reside. She believes there are about fourteen. Can the editor or readers of the "Mirror" kindly help her?

If this question refers to medical officers of health the information can be obtained from Churchill's "Medical Directory," in which, under the heading "Sanitary Medical Service," the names of all medical officers of health are given.

*Date of Article.*

(171) Nurse C. would be obliged if the editor would let her know the date of issue containing the account of the opening of the new Nurses' Home, Great Ormond Street.

The number of the "Mirror" containing the article in question is March 25th, 1899.

*Midwifery Department.*

(172) Would you kindly tell me whether a superintendent nurse, knowing absolutely nothing about midwifery or maternity nursing, should interfere with the lying in ward of the workhouse to which she has been appointed; that ward being under the charge of two certified midwives?—*One in Difficulty.*

The duties of the superintendent nurse of a workhouse are defined by the Committee of Management, to whom all such questions as the above should be referred.

*Mission Nursing in Africa.*

(173) The mission that applied for nurses about six months ago was the Universities' Mission to Central Africa. Applicants should apply to the Rev. Duncan Travers, 9, Dartmouth Street, Westminster, S.W.

### Travel Notes.

By Sister Grace.

### XXXI.—INTERLAKEN.

Almost everyone wishes to go to Switzerland, and there are charms in that favoured land not to be found in any other country. There are, however, two drawbacks—first, it is overrun with tourists, funicular railways, restaurants in every corner where they should not be, and touts of every description. All this popularity makes living expensive in the season, which lasts from the end of July to the beginning of October. Secondly, the journey is of necessity dear. Still, in the case of nurses, a little determination to save during the year will overcome the difficulty, and I am sure that the pleasure received will be very great.

### The Expenses of the Journey.

Second-class return *viâ* Dieppe and Paris is £5 7s. 7d., and the ticket holds good for forty-five days, if you are lucky enough to be able to remain so long. Only one night is spent in the train, and you will arrive at half-past one on the day after you leave London. There is an hour's wait at Pontarlier, which gives time for a wash, rest, and breakfast. If it is necessary to study severe economy avoid the expense of breakfast by taking food with you.

### Where to Stay.

If you do not want to be fashionable and you do wish to do your holiday cheaply go to the Hotel du Lac, close to the East Station (very convenient). Here you may live for six francs per day ; or to the Hotel Untersen on the same terms. The latter is in Untersen itself, which is the oldest and most picturesque part of Interlaken. If you have a long time to stay in Switzerland you can have still cheaper accommodation in the environs of Interlaken, but for a short stay it is not worth while.

### Position of Interlaken.

This, as its name indicates, is between two lakes, those of Brienz and Thun, and this it is, I think, which gives its peculiar charm ; the steamers are very cheap, and when one is too idle to do anything else one can always step on board and be carried near or far as the spirit moves one. Both lakes are lovely. I prefer Brienz myself, but must not stop to speak of that now ; it deserves a paper to itself. The *trajet* from Interlaken to Thun is delightful, and the little old-world town of Thun itself is charming. The principal street on market day is a wonderful sight with booths for every kind of merchandise, notably for the two kinds of pottery for which Thun is famous. Never buy this in the shops, where it is much more expensive. On each side of the street are raised walks, under which are booths and over are shops. At the end of this street there are some fine municipal buildings, and high above, attained by a picturesque pitched walk or innumerable steps, stands the old castle of Zähringen-Kyburg, erected 1429. Close by is the church, and from all corners of this elevated plateau the views are splendid, especially of the Bernese Oberland.

### Interlaken Itself.

It is a very gay little town in the season. Hotels, shops, and promenades swarm with pleasure-seekers. On the road between the landing-stage of the Brienz boat and the town there is the celebrated view of the Jungfrau. It lies straight before our entranced eyes, shooting up between a cleft in the nearer mountains, dazzlingly white and pure. In early spring the effect is most enthralling, when the foreground is a rich half meadow, backed by brilliant green larches, and the middle distance of dark firs is thrown into unusual prominence.

### The Old Town.

For artists and photographers the old part called Untersen abounds in charms. The church, with its characteristic little campanile, is delightful outside, but, alas ! when the seeker after architectural treasures enters, what a chilling disappointment ! Like all Zwinglian churches, it is bare, cold, and empty, and one hurries out lest one should perish of arctic cold both bodily and spiritually. The Hotel Untersen is in the old square with the church, the fountain, and several charmingly picturesque old houses. Here, the artist will revel and find some difficulty in tearing himself away for the numerous excursions, of which I must speak another day.

### Hints to Travellers.

When economy is imperative it is advisable to take your food with you, for it is invariably expensive at station buffets and often very unappetising. But be careful what you take. In warm weather avoid butter and milk ; cold tea is more refreshing than anything ; make it fresh immediately before you start, do not allow it to stand more than two minutes, and pour it into a wine bottle. Take penny rolls instead of slices of bread, they do not become so dry ; no butter, but fruit, oranges, bananas, apples, plums, &c.—not strawberries or raspberries, both bad travellers. If weather permits veal pies are suitable things, the crust of which they are made being substantial and not flaky ; hard-boiled eggs are good too ; if sandwiches are taken they should be eaten at the first meal, or they will become dismally wrinkled and curled.

---

### TOURS FOR THE INEXPERIENCED.

Any nurses wishing to pay a short visit to Switzerland, but feeling alarmed at the responsibilities of travel, would do well, I think, to avail themselves more than they do of Dr. Lunn's parties. He has several starting in August and September. The expense for a nine days' stay in Geneva and journey inclusive is only £6 6s. ; for twelve days the cost is £8 8s., including three days at Chamonix. For those who have never been abroad before and feel a little nervous these tours are the very thing and astonishingly cheap, as you will acknowledge when you remember that the journey alone to Chamonix and back is £5 16s. 3d.

---

### TRAVEL NOTES AND QUERIES.

Rules in Regard to Correspondence for this Section.—All questioners must use a pseudonym for publication, but the communication must also bear the writer's own name and address as well, which will be regarded as confidential. All such communications to be addressed "Travel Editor, 'Nursing Mirror,' 28, Southampton Street, Strand." No charge will be made for inserting and answering questions in the inquiry column, and all will be answered in rotation as space permits. If an answer by letter is required, a stamped and addressed envelope must be enclosed, together with 2s. 6d., which fee will be devoted to the objects of the "Hospital Convalescent Fund." Any inquiries reaching the office after Monday cannot be answered in "The Mirror" of the current week.

Dolce Acqua (Florizel).—I think you must have mistaken the name. Dolce Acqua is a very small mountain town, now little more than a village, on the Riviera, behind Ventimiglia and Bordighera. No accommodation to be had there, as far as I know, but quite attainable from Bordighera. Write me again if I can help.

Veules (Short Holiday).—This is a cheap and pleasant little place reached by omnibus from St. Valery-en-Caux. It is quiet, but as you say you want to read for an examination I think it would suit you, and as you are a bicyclist you could penetrate inland along the many delightful Norman roads, which are ideal for the cyclist. There are two hotels, but, being a bachelor, I think you could manage much more cheaply in a lodging, which you are sure to find in the little place.

S. Beatenberg (Perseus).—Second class return to Interlaken, £5 7s. 7d. ; thence by cable railway or road. Living fairly cheap if you are content with the humbler hotels. Hotel Pension Beau Sejour, seven to ten francs ; Pension Silverhorn, six to ten francs ; Hotel Pension des Alpes, five to six francs. It is somewhat overrun with tourists now, and has quite lost the simplicity of a few years ago.

Tintagel (Devonian).—Yes, the new hotel is open, but I have not tried it. I prefer the old-fashioned Wharncliffe Arms. It is quite possible to visit Tintagel from Boscastle in a day, but you would enjoy a week there if time permits. As a centre for excursions Boscastle is preferable, because Tintagel is four miles from a station.

Sketching (Artist).—The water-colour box you want, with rest attached, is to be had of Messrs. Reeves and Sons. The price I do not know.

## Figures and Tables

**Figure f1:**